# The complete chloroplast genome of a mangrove *Kandelia obovata* Sheue, Liu & Yong

**DOI:** 10.1080/23802359.2019.1674713

**Published:** 2019-10-07

**Authors:** Zhaokui Du, Junmin Li, Dang Yang

**Affiliations:** a Zhejiang Provincial Key Laboratory of Plant Evolutionary Ecology and Conservation, Taizhou University, Taizhou, Zhejiang, P. R. China;; b Institute of Ecology, Taizhou University, Taizhou, Zhejiang, P. R. China

**Keywords:** *Kandelia obovata*, complete chloroplast genome, phylogenetic analysis

## Abstract

*Kandelia obovata* is a widely distributed species of mangrove in Eastern Asia. In this study, the complete chloroplast genome sequence of *K. obovata* was assembled and characterised from high-throughput sequencing data. The chloroplast genome was 168,244 bp in length, consisting of large single-copy (LSC) and small single-copy (SSC) regions of 94,869 bp and 20,088 bp, respectively, which were separated by a pair of 26,618 bp inverted repeat (IR) regions. The genome is predicted to contain 129 genes, including 84 protein-coding genes, 37 tRNA genes, and 8 rRNA genes. The overall GC content of the genome is 34.6%. A phylogenetic tree reconstructed by 15 chloroplast genomes reveals that *K. obovata* is mostly related to *Rhizophora stylosa.*

Mangroves are a group of salt-tolerant trees and shrubs belong to different families that live in the intertidal regions of the tropical and subtropical coastlines (Du and Li 2019). *Kandelia obovata* is a widely distributed species of mangrove in Eastern Asia (Sheue et al. 2003). It is well known that the latitudinal distribution of mangroves is mainly limited by temperature (Wang et al. 2011); however, among all of the mangrove species, *K. obovata* has the highest cold tolerance and becomes a dominant coastal mangrove species in China (Chen et al. 2017). It was successfully introduced to Yueqing Bay in Zhejiang Province (28°20′ N) from its naturally northernmost distribution site in Fuding, Fujian Province (27°17′ N) in 1950s (Su et al. 2019). In addition suffering from frozen in winter, *K. obovata* is threatened by aquaculture expansion, deforestation, pollution, UV radiation enhancing, sea level rising, and natural calamities (Guan et al. 2015), so the populations have been dramatically decreased in recent decades and urgently need protection and restoration, especially in higher latitude area. Previous studies on *K. obovata* mainly focussed on population genetic diversity; the adaptation and tolerance mechanisms to flooding, cold, salt, heavy metal, and organic pollutant involving antioxidant enzymes, gas exchanges, and the expression profile of the response-associated genes and proteins (Pi et al. 2017; Pan et al. 2019; Su et al. 2019). However, there have been still no reports about chloroplast genome information of *K. obovata* yet. In this study, the complete chloroplast genome of *K. obovata* using high throughput sequencing technology was determined, which will provide informatics data for the phylogeny of *Kandelia* genus and further research on *K. obovata*.

The fresh leaves of *K. obovata* were sampled from Yuhuan, Zhejiang, China (28°13′ N, 121°10′ E). Specimens were stored in the Herbarium of Taizhou University (accession number: TZU-20190525KO01). Total genomic DNA was extracted from the fresh leaves with a modified CTAB protocol according to Doyle and Doyle (1987). The whole genome sequencing was conducted by Hefei Biodata Biotechnologies Inc. (Hefei, China) on the Illumina Hiseq platform. The filtered sequences were assembled using the programme SPAdes assembler 3.10.0 (Bankevich et al. 2012). Annotation was performed using the DOGMA (Wyman et al. 2004) and BLAST searches.

The cp genome of *K. obovata* was determined to comprise a 168,244 bp double-stranded, circular DNA (GenBank accession no. MN117072), which containing two inverted repeat (IR) regions of 26,618 bp, separated by large single-copy (LSC) and small single-copy (SSC) regions of 94,869 bp and 20,088 bp, respectively. The overall GC content of *K. obovata* cp genome is 34.6% and the corresponding values in LSC, SSC, and IR regions are 31.9, 28.1, and 42.0%, respectively. The cp genome was predicted to contain 129 genes, including 84 protein-coding genes, 37 tRNA genes, and 8 rRNA genes. Seven protein-coding genes, eight tRNA genes, and four rRNA genes were duplicated in IR regions. Nine genes contained two exons and four genes (clpP, ycf3, and two rps12) contained three exons.

To investigate its taxonomic status, alignment was performed on the 15 chloroplast genome sequences using MAFFT v7.307 (Katoh and Standley 2013), and a maximum likelihood (ML) tree (*Ficus racemosa* were used as the outgroup) was constructed based on 63 single-copy gene by FastTree version 2.1.10 (Price et al. 2010). As expected, *K. obovata* is mostly related to *Rhizophora stylosa* with bootstrap support values of 100% (Figure 1). The complete cp genome sequence of *K. obovata* will provide a useful resource for the conservation genetics of this species as well as for the phylogenetic studies of *Rhizophoraceae*.

**Figure 1.**
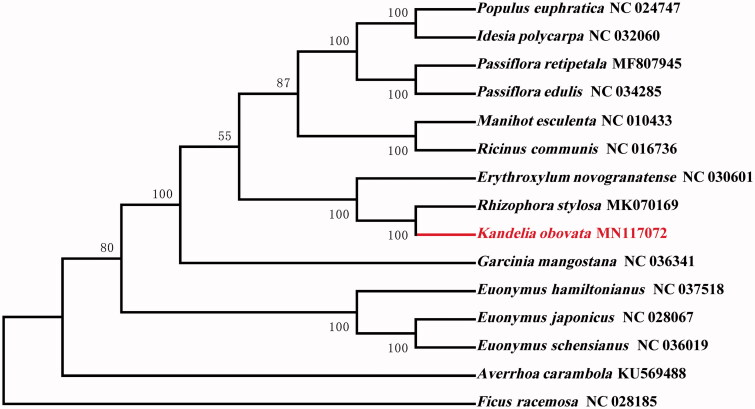
Phylogenetic tree inferred by maximum likelihood (ML) method based on 63 single-copy gene of 15 representative species. *Ficus racemosa was* used as an outgroup. A total of 1000 bootstrap replicates were computed and the bootstrap support values are shown at the branches. GenBank accession numbers were shown in Figure 1.
